# Constructing a Global Learning Partnership in Physiotherapy: An Ireland–Uganda Initiative

**DOI:** 10.3389/fpubh.2017.00107

**Published:** 2017-06-12

**Authors:** Cliona O’Sullivan, Herman Kazibwe, Zillah Whitehouse, Catherine Blake

**Affiliations:** ^1^School of Public Health, Physiotherapy and Sports Science, University College Dublin, Dublin, Ireland; ^2^Department of Physiotherapy, Mbarara University of Science and Technology, Mbarara, Uganda

**Keywords:** global health, physiotherapy, disability, rehabilitation, curricula, collaborative learning, partnership

## Abstract

**Background and aim:**

There is a strong correlation between disability and poverty and it is acknowledged that until disability issues are addressed, the goal of poverty reduction in low-income countries is unlikely to be achieved. Despite the high prevalence of disability in developing countries, there remains a significant shortage of rehabilitation professionals as highlighted by the WHO report, Human resources for Health (2006). The purpose of this project was to develop a collaborative and sustainable partnership to strengthen educational and research capacity in global health, disability, and rehabilitation between two physiotherapy schools; University College Dublin, Ireland, and Mbarara University of Science and Technology, Uganda. This article aims to describe the approach used and initial project outcomes.

**Methods:**

This project involved a bilateral visit to both institutions by two members of staff of respective physiotherapy programs. These visits entailed stakeholder meetings, clinical site visits, and workshops to identify the priorities for the partnership and shape the collaboration going forward. Appreciative inquiry methodology was used during the workshops and the four-dimensional framework for curriculum development was used to guide analysis and underpin findings.

**Findings:**

The key priorities identified were (i) development of joint global health learning initiative, (ii) to explore the possibility of postgraduate learning and research opportunities for Ugandan colleagues, and (iii) to develop joint clinical placements. The rationale and context and a plan of action is described.

**Discussion and conclusion:**

The project is ambitious and in order to be sustainable, the importance of long-term interinstitutional commitment and further funding cannot be ignored. This work provides a framework for other universities and institutions wishing to undertake similar activities. Such partnerships provide rich learning opportunities for students and health professionals and facilitate a deeper understanding of global health issues, social and cultural health determinants, and development of enhanced professional skills.

## Introduction

Eighty percent of the world’s disabled population lives in developing countries (1). There is a strong correlation between disability and poverty and it is acknowledged that until disability issues are addressed, the United Nations (UN) sustainable development goal of poverty reduction is unlikely to be achieved (1, 2). Child disability research in low-income countries highlights the significant negative impact of disability on families, particularly women, financially, educationally, psychologically, and culturally (3, 4).

The recent World Health Organization report (5), Human Resources for Health, highlights the chronic shortage of health workers, particularly in developing countries. Most of the available evidence relates to numbers of nurses, midwives, or physicians per 10,000 population, with a dearth of information regarding health professionals working in the area of disability and rehabilitation, such as physiotherapists.

Physiotherapists are an integral part of the multidisciplinary team that serves to prevent disability and maximize function and quality of life. The World Confederation for Physical Therapy (WCPT) states that: “Physical therapy is concerned with identifying and maximizing quality of life and movement potential within the spheres of promotion, prevention, treatment/intervention, habilitation, and rehabilitation. This encompasses physical, psychological, emotional, and social well-being.” It is a discipline that lends itself well to being practiced in low resource settings.

The prevalence of disability in Uganda is estimated to be between 16% and 19% (6, 7). The World Report on Disability (1) estimates that there are 0.25 physiotherapists per 10,000 population in Uganda compared to 5 per 10,000 in the United Kingdom, a statistic which Ugandan physiotherapists consider an overestimation (personal communication). The report also advocates for the support of universities in developed countries to build and strengthen educational and research capacity by supporting training programs in developing countries and developing research partnerships.

The purpose of this project was to develop a collaborative and sustainable partnership to strengthen educational and research capacity in global health, disability, and rehabilitation between two physiotherapy schools; University College Dublin (UCD), Ireland and Mbarara University of Science and Technology (MUST), Uganda. This article aims to describe the approach used and initial project outcomes.

## Methods

### Purpose

The overarching vision for this project was to develop a collaborative and sustainable partnership between the discipline of physiotherapy in UCD and MUST. Initial discussions took place between COS and HK via e-mail and Skype to discuss the philosophy and rationale for the partnership, potential mutual benefits, and feasibility. The philosophy underpinning the project was that all physiotherapy graduates should have knowledge and understanding of global health issues and an awareness of different health systems. It was recognized that the partnership would have mutual and bilateral benefits to students and staff of both universities in terms of knowledge transfer and skills development. Finally, it was recognized that good communication and commitment were key to the success of the partnership. Through this project, the authors aimed to generate a framework which will facilitate collaboration and transfer of knowledge, understanding, experience, and research skill, between UCD and MUST physiotherapy students and academic staff regarding global health, disability, and rehabilitation.

### Setting

Mbarara University of Science and Technology is the second public university in Uganda founded in 1989. It has since grown to have 3,000 students over 6 faculties and 2 institutes (Faculty of Medicine, Faculty of Applied Science, Institute of Management Science, Institute of Computer Science, Institute of Interdisciplinary Training and Research, Institute of Forestry Management and Institute of Child and Maternal Health). The Faculty of Medicine trains students in medicine, nursing, medical laboratory science, pharmacy, pharmaceutical science, physiotherapy and counseling at a variety of diploma, degree, and postgraduate levels. The vision of the Faculty is “to be recognized as a center of excellence in health sciences education, research, and community service.”

The BSc Physiotherapy Course at MUST was envisioned by staff in the neighboring government hospital in 2007 and started in 2012. It is a 4-year degree program and currently has 60 students. The program is expected to grow to 100 students over the next 5 years. The first pioneer students completed their degree in June 2016. The mission of the Department of Physiotherapy is “To provide high-quality evidence-based education in Physiotherapy with emphasis on producing innovative, research orientated students who will translate the knowledge and skills into practices that will improve the health and well-being of the nation and beyond.”

University College Dublin is Ireland’s largest university and is ranked in the world’s top 100 university in clinical, preclinical, and health sciences. Physiotherapy at UCD is based in the multidisciplinary School of Public Health, Physiotherapy, and Sports Science. The physiotherapy program at UCD is more than 60 years old and it is the largest provider of undergraduate and graduate physiotherapy education in Ireland. There are two entry-to-practice level programs in physiotherapy: a 4-year BSc physiotherapy program and a 2-year professional master of physiotherapy program. It also offers a range of postgraduate programs for physiotherapists.

### Background to Collaboration

Similar to many health professions, there is a strong tradition of Irish physiotherapy students choosing to undertake clinical elective placements in low and middle income countries and indeed working in developing countries after graduation. Building on this tradition, in 2013, together with a UCD graduate physiotherapist who was working in Uganda, an international elective clinical placement was developed in conjunction with the university charity, UCD Volunteers Overseas (UCDVO) in Kisiizi Hospital, a rural hospital in the Rukungiri district of South Western Uganda, one of the nine priority countries of Irish Aid.

At the same time, MUST was building links with Kisiizi Hospital through a physiotherapy colleague who had met some of the visitors from Ireland and recommended that a link would be positive. From there subsequent meetings were arranged. As the physiotherapy department was new, the faculty at MUST was also recommending the establishment of international links.

The link between Ireland and Uganda was further strengthened in 2015, during a visit to Mbarara University of Science and Technology to explore the possibility of developing a partnership between the two Schools of Physiotherapy. In line with recommendations from the International Health Links Manual (8), the schools of physiotherapy at UCD and MUST share a mission to produce high-quality graduates who are active citizens and can adapt and lead in challenging health care environments. Training for rehabilitation and other health professionals in developing countries is complex due to the need for graduates to work independently with limited resources and often without support services (1). In addition, the World Report on Disability, advocates for the support of universities in developed countries to build and strengthen educational and research capacity by supporting training programs in developing countries and developing research partnerships (1). Between 2015 and 2016, physiotherapy staff at both UCD and MUST have worked together via e-mail and telephone to develop this proposal for funding which would facilitate the establishment of a partnership between the two schools. The project was awarded funding in 2016 from the ESTHER Alliance (http://www.esther.ie/).

### Project Structure

This project involved bilateral visits to both institutions by two members of staff of each of the respective physiotherapy programs. These visits entailed general discussion with a variety of stakeholders including students, academic and clinical staff, and clinical site visits, in order to gain a deeper appreciation of the context and priorities of the respective health systems, the practice of physiotherapy, and the potential for physiotherapy to meet the current and future health needs of the respective communities, in a multidisciplinary context. At the end of both visits, a structured workshop using an appreciative inquiry (AI) methodology was undertaken. The purpose of the structured workshops was to harness the knowledge and experience of academic and clinical colleagues to best identify the priorities for the partnership and shape the collaboration going forward.

**Figure 1:**
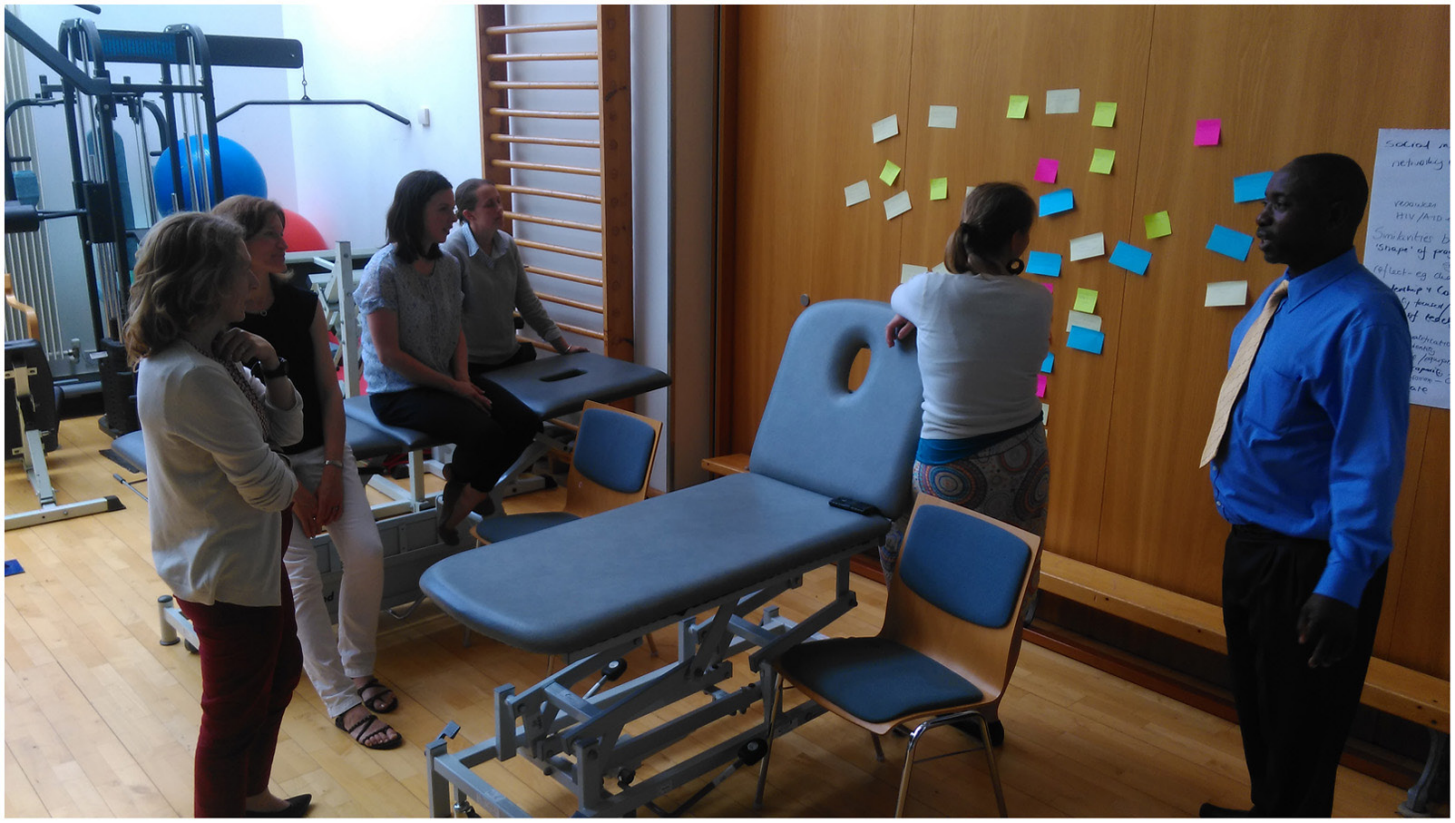
**Workshop conducted at UCD**.

**Figure 2:**
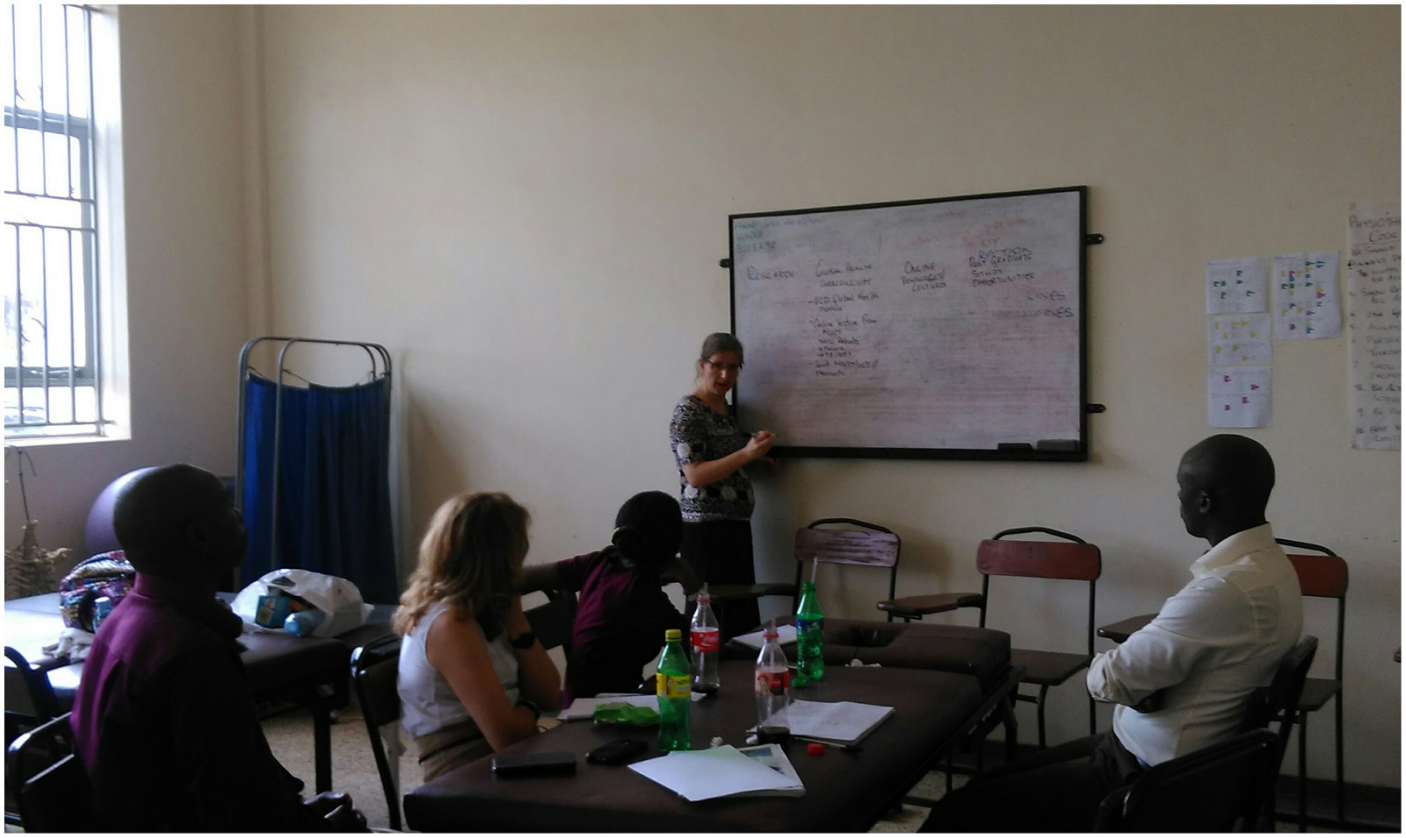
**Workshop conducted at MUST**.

The project was conducted in two phases. Phase 1 took place at UCD between June 6 to June 10, 2016 and Phase 2 took place in Uganda from July 4 to July 10, 2016. Both visits had a similar schedule with workshops being conducted in the respective physiotherapy schools both in Dublin and Uganda (Figures 1 and 2). The final workshop in Uganda focused on identifying the key priorities for the partnership and designing a plan of action in relation to same.

A method known as AI was used to achieve the goal of each workshop. AI has been used in organizational change management, education, and healthcare settings. It is a qualitative approach that adopts a positive and strengths-based approach to maximize a project’s potential (9). Contrary to “traditional” problem solving approaches, where the “problem” or deficit is central, key to this strengths-based appreciative approach is valuing the best of what exists in the present and envisioning the potential based on existing strengths. Core to the concept is rather than asking “what is wrong” in a particular case, project or organization, the focus is on “What is good?” It is proposed that using this model promotes collective strengths to problem solve and envisage a better situation in the future (9).

Each workshop (Ireland and Uganda) was conducted in four steps, in line with the AI approach: discover, dream, design, and deliver. Participants in the workshop in Ireland included UCD physiotherapy academic staff, clinical tutors, and visiting faculty from Uganda (*n* = 9). In Uganda, participants were MUST physiotherapy academic staff and visiting faculty from Ireland (*n* = 6). Each workshop began with a brief presentation by the facilitators regarding the overarching project goal, and the rationale for and structure of the workshop. The visitors also presented an overview of their country, health system, physiotherapy practice, school information, and curricula.

#### Step 1: Discover

The objective of this step was to allow the participants to learn about the visiting institution but also to reflect and relate personal experiences and opinions. Open discussion across a broad range of topics was encouraged. Participants were encouraged to ask questions and share experiences. Questions used to prompt discussion and reflection included:

- What have you learned/“discovered” about the visiting institution?
- What is your experience with collaborations and partnerships? What worked well/what didn’t work well?
- Why/how has this process/experience occurred?
- Is this process/experience important/valuable? If so, why?
- What else would you like to know?

#### Step 2: Dream

The objective of Step 2 was to create a vision for the partnership with “blue skies thinking.” Groups were seated in a circle and each participant had a card upon which they wrote as many ideas as they could in 1 min. They then passed the card to the next person, who reviewed their statement and added more of their own. The rationale is that ideas and thoughts are generated and strengthened as they are peer-reviewed.

The group then reviewed all statements and emerging topics were grouped thematically. Each theme was then discussed and group consensus was used to determine whether it was a priority for the partnership or not.

#### Steps 3: Design

Once key priorities were agreed upon, the group planned processes that would work well to achieve the desired priority.

#### Step 4: Deliver

Finally, the group discussed how the proposed design would be implemented and plans of action were developed.

### Theoretical Framework

The 4DF approach was used as a theoretical framework on which to guide our analysis and describe our findings. This framework allows the relationship between curricula and bigger issues that shape health professional education practice and policy to be explored (10). This conceptual framework suited the project needs, as it facilitated the development of a learning partnership which considered the differences, as well as the similarities, across both programs and socio-cultural, economic, and environmental contexts. The four curriculum dimensions are described (10): Dimension 1: identifying future healthcare practice needs; Dimension 2: defining and understanding capabilities; Dimension 3: teaching, learning, and assessment; and Dimension 4: supporting institutional delivery.

## Findings

### Dimension 1: Identifying Future Health Care Practice Needs

#### Burden of Disease

The priorities for physiotherapy practice across both countries are different and reflect the burden of disease that is unique to each country. Current WHO data reveal that the leading causes of death in Ireland are ischemic heart disease, stroke, and lung cancer, while in Uganda the leading causes of death are HIV/AIDs, lower respiratory tract infections, and malaria, (11). Non-communicable diseases are main contributors to the burden of disease in Ireland while in Uganda, HIV, TB, malaria, and neonatal conditions are significant contributors to the burden of disease as measured by disability-adjusted life years (11). An increase in the aging population in Ireland poses further challenges while in recent years, the increase in non-communicable disease such as ischemic heart disease and stroke observed in Uganda highlight the growing double burden of both non-communicable and infectious diseases in many low-income countries.

The increase in non-communicable diseases observed in recent decades is directly related to lifestyle and non-invasive and non-pharacological interventions such as physiotherapy have been demonstrated to be highly effective in preventing and managing such conditions (12).

#### Evidence-Based Practice

Recently, disability has been placed centrally on the development agenda with the publication of the World Disability Report in 2011 and also by its inclusion in the sustainable development goals. The link between poverty and disability is well established and it is acknowledged that until disability issues are addressed, the global goal of poverty reduction will not be achieved. There is therefore an opportunity for physiotherapists to lead to advocate for disability awareness and inclusion, disability services and research across the health, education, and social spectrum.

While there is significant evidence to support physiotherapy in the prevention and management of chronic disease, less is known about the impact of physical rehabilitation on many of the conditions encountered by physiotherapists working in low-income settings such as HIV/AIDs and cerebral palsy (sometimes as a result of cerebral malaria). Small-scale qualitative studies have highlighted the positive impact of rehabilitation on patients with HIV/AIDs (13) and children with cerebral palsy (3, 14). However, larger scale longitudinal studies are necessary.

There is now an opportunity to collaborate on and strengthen chronic disease research (stroke, diabetes, ischemic heart disease, low back pain) that is currently being undertaken in UCD and MUST. Therefore, graduates from both institutions will have robust research skills so that the evidence base for physiotherapy in a global context can be strengthened.

### Dimension 2: Defining and Understanding Capabilities

The physiotherapy curricula at both UCD and MUST adhere to the guidelines for entry-level physical therapy education as set out by the WCPT and are accredited by the Irish Society of Chartered Physiotherapists and the Allied Health Professions Council in Uganda respectively. Therefore, both curricula have strong similarities, with themes such as health promotion, physical activity, and exercise emerging strongly. Professional practice and interprofessional learning was also key to both programs, particularly at MUST, where educators developed a 10-week interdisciplinary community placement during every recess term, where students from all the healthcare disciplines work together in a rural community setting for holistic health delivery. Furthermore, the Department of Physiotherapy at MUST delivers a module on rehabilitation medicine to both medical and nursing students so that these health professionals have an awareness of disability issues and the rehabilitation of the same. The importance of interprofessional learning to enhancing patient care and health outcomes has been highlighted by the WHO (15) and is increasingly part of health professions curricula by accrediting bodies (16).

#### Clinical Education

Clinical education is a significant component of both programs with all students completing a minimum of 1,000 h of clinical education. Clinical education is key to all health professions programs: it is here that theory is translated to practice and development of professional competencies and behaviors come to the fore.

There is a value to participating on clinical placement with students and staff from other countries. It leads to greater understanding of respective health systems, health challenges and allows a deeper understanding of respective cultures and belief systems and their influence on health.

The value of overseas placements to health professionals is well established with some health systems actively creating opportunities for staff to participate in overseas programs because of the tangible benefits to their staff including: development of soft skills such as communication awareness, leadership, and teamworking skills (17). Similar benefits are observed in studies of students undertaking placements overseas (18), including physiotherapy students (19).

### Dimension 3: Teaching Learning and Assessment

#### Education Resources

Differences existed in terms of the physical and online resources available, particularly access to online library resources and other information resources which are restricted in Uganda. However, social media (Twitter, Facebook, and What’s App) was used extensively in Uganda as a means of sharing information and drawing students’ attention to useful information or events. There was an opportunity to share learning resources and this has already begun in an informal manner. Lecturers have agreed to facilitate recorded lectures or lectures/tutorials via Skype.

#### Development of Joint Global Health Learning Initiative

There was agreement that the collaboration provided an opportunity to establish and develop a joint global health learning initiative. This was deemed a rich learning opportunity for students from both programs to improve knowledge and understanding regarding global health and disability issues and the link between disability and development. It would also serve to improve student knowledge regarding cultural influences as a determinant of health.

#### Postgraduate Study Opportunities for Staff

The lack of postgraduate study opportunities for physiotherapists in Uganda was highlighted across all areas of physiotherapy practice. A member of faculty is interested in pursuing postgraduate study in the area of cardiorespiratory physiotherapy and funding opportunities will be explored. This is deemed important considering the rise in the burden of non-communicable diseases particularly ischemic heart disease, stroke, and cancers.

### Dimension 4: Supporting Institutional Delivery

Dimension 4 addresses the aspect of cultural norms, protocols, and procedures inherent within the educational context. During both visits, the visiting lecturers had the opportunity to meet stakeholders from disciplines such as development studies, medicine, nursing, dietetics, and public health. There was agreement that there were tangible benefits related to teaching and learning and research, and there was support for formal agreement being developed between the two departments. The partnership is also aligned with university strategies. Discussion also took place about the opportunity to embed this work and collaborate with external agencies such as international non-governmental organizations.

### Key Priorities

At the end of the workshop in Uganda, three priorities were identified and action plans were developed. Two members of faculty at each institution will lead on realization of the identified priorities (UCD-COS, CB, MUST-HK, and ZW).

#### Priority 1: Develop a Joint Global Health Learning Initiative for Entry-Level Physiotherapy Students in Uganda and Ireland

##### Plan of Action

Physiotherapy graduates from both institutions will need to have the skillset required to identify and respond to current and future health care practice needs across different contexts. To facilitate a greater understanding of health systems, burden of disease, and physiotherapy priorities for both countries, it was proposed that students from both programs will work together on discrete learning activities. The learning activities have two goals: (i) to develop a greater awareness of a different health system and (ii) to share knowledge and understanding in terms of physiotherapy practice. This will be realized by developing case studies that are relevant to both student groups (e.g., cerebral palsy, stroke, HIV/AIDs). This approach was developed as a initiative of Oxford Brookes University and Mbarara School of Physiotherapy was one of the pioneering participants. It is proposed that student groups will work together to present the relevant social context and how the case is managed within their health system. Use of technology, online learning resources and social media will be harnessed to promote collaborative and shared learning across the groups. Examples may include wikis, blogs, and social media. Students will present their final work via video link to their peers on both programs. Achievement of this activity will be dependent on internet connectivity and cost issues.

Both programs are accredited by their respective national accreditation bodies. This learning activity will be facilitated by the project leads (COS, HK) and will be embedded in relevant modules and therefore part of accreditation submissions. We believe that the proposed global learning partnership will strengthen curricula, particularly in the development of soft skills such as communication, teamwork, cultural competency, the development of which are an integral part of accreditation standards. The effectiveness of this educational activity in developing these soft skills will be evaluated in future studies.

#### Priority 2: Explore the Possibility of Postgraduate Learning and Research Opportunities for Ugandan Colleagues

##### Plan of Action

A pathway for postgraduate study in cardiorespiratory physiotherapy will be created in UCD and scholarship funding will be sought jointly. Funding opportunities for joint PhD research in the area of physiotherapy and rehabilitation will be explored and proposals developed. In addition, the opportunity for joint undergraduate research projects will be explored, particularly comparative studies between the two countries. Each of these serve to strengthen the evidence base for physiotherapy, particularly in low-income settings.

#### Priority 3: Establish Joint Clinical Placements for Entry-Level Physiotherapy Students in Uganda and Ireland

##### Plan of Action

Since 2015, a small number physiotherapy students from both UCD and MUST (three from each university) have participated in a joint clinical placement in a rural hospital in Uganda. For UCD students, this is part of the curriculum while for MUST students it is an extracurricular activity. This initiative will be expanded to include community settings. The opportunity to develop this as an interprofessional placement will also be explored, to include UCD medical, nursing, and dietetics students working together with students from MUST, to engage with local communities and experience health systems in developing countries. The experience of students participating in this initiative will be evaluated during the coming year.

## Discussion

This article aims to describe an approach used to establish a global learning partnership to strengthen educational and research capacities in global health, disability, and rehabilitation between two physiotherapy schools; UCD Physiotherapy and the Department of Physiotherapy at MUST.

The main priorities identified were aligned to the 4DF approach and are (i) develop a joint global health learning initiative, (ii) to explore the possibility of postgraduate learning and research opportunities for Ugandan colleagues, and (iii) to develop joint clinical placements.

The 4DF approach facilitated a comprehensive description and presentation of the key priorities for the partnership. It has also allowed clear alignment of activities and outputs for each priority. As this project develops, the 4DF approach will continue to be used to identify and monitor achievements and milestones. An evaluation of the activities undertaken will be presented in future studies.

Developing this partnership has the potential to affect both educational (short term) and health outcomes (long term). The partnership will improve student knowledge and understanding regarding global health and disability issues, cultural influences on health, and awareness of strategies for management of minority, migrant, and “hard to reach” groups. It will foster and develop transferable skills in students, such as communication, professionalism, cultural competency, leadership, and advocacy skills. It will facilitate the development of sound research skills among students in the area of disability and global health and increase opportunities for students to pursue MSc and PhD level education.

The improved quality of education resulting from this partnership will translate to improvement in functional capacity and quality of life for patients with disabilities and their carers, as a direct result of enhanced quality of rehabilitation. The partnership will strengthen the evidence base for rehabilitation in both low- and high-income countries, improving quality of care.

There are many examples of existing partnerships between health science schools in high- and low-income countries (8) and the benefits for both partners is well established (8). This work adds to the literature and provides a framework for universities and institutions to guide the development and growth of such partnerships.

The project is ambitious and in order to be sustainable, the importance of long-term inter-institutional commitment and further funding cannot be ignored. This article seeks to describe the initial steps taken in establishing a partnership and determining common priorities for the partnership going forward. There are many challenges that must be overcome if the priorities identified will be realized. These included sustained commitment between the two institutions. To date, this has been achieved through regular Skype meetings and e-mail contact. Funding opportunities for travel, postgraduate study, and research scholarships must be explored and acquired. Finally demands on time and internet connectivity provide challenges.

To conclude, partnerships such as this can provide rich learning opportunities for students and health professionals and facilitate a deeper understanding of global health issues, social, and cultural health determinants and development of enhanced professional skills. Future work will focus on the implementation and evaluation of the priorities identified during this initial stage, therefore building the evidence base for global learning partnerships.

## Author Contributions

COS and HK developed the original proposal. All authors contributed to the design, implementation, analysis, and write-up. COS led the submission of the manuscript.

## Conflict of Interest Statement

The authors declare that the research was conducted in the absence of any commercial or financial relationships that could be construed as a potential conflict of interest.
